# Diagnostic Validity of High-Density Barium Sulfate in Gastric Cancer Screening: Follow-up of Screenees by Record Linkage with the Osaka Cancer Registry

**DOI:** 10.2188/jea.JE20090116

**Published:** 2010-07-05

**Authors:** Kenyu Yamamoto, Hideo Yamazaki, Chikazumi Kuroda, Tsugio Kubo, Akira Oshima, Toshizo Katsuda, Tadao Kuwano, Yoshihiro Takeda

**Affiliations:** 1 Department of Radiological Technology, Graduate School of Health Sciences, Okayama University, Okayama, Japan; 2 Department of Radiology, Osaka Cancer Prevention and Detection Center, Osaka, Japan; 3 Cancer Information Services, Osaka Medical Center for Cancer and Cardiovascular Diseases, Osaka, Japan; 4 Faculty of Health Care Sciences, Himeji Dokkyo University, Himeji, Hyogo, Japan

**Keywords:** gastric cancer screening, barium sulfate, sensitivity, specificity, area under ROC curve (AUC) analysis

## Abstract

**Background:**

The use of high-density barium sulfate was recommended by the Japan Society of Gastroenterological Cancer Screening (JSGCS) in 2004. We evaluated the diagnostic validity of gastric cancer screening that used high-density barium sulfate.

**Methods:**

The study subjects were 171 833 residents of Osaka, Japan who underwent gastric cancer screening tests at the Osaka Cancer Prevention and Detection Center during the period from 1 January 2000 through 31 December 2001. Screening was conducted using either high-density barium sulfate (*n* = 48 336) or moderate-density barium sulfate (*n* = 123 497). The subjects were followed up and their medical records were linked to those of the Osaka Cancer Registry through 31 December 2002. The results of follow-up during 1 year were defined as the gold standard, and test performance values were calculated.

**Results:**

The sensitivity and specificity of the screening test using moderate-density barium sulfate were 92.3% and 91.0%, respectively, while the sensitivity and specificity of the high-density barium test were 91.8% and 91.4%, respectively. The results of area under receiver-operating-characteristic (ROC) curve analysis revealed no significant difference between the 2 screening tests.

**Conclusions:**

Screening tests using high- and moderate-density barium sulfate had similar validity, as determined by sensitivity, specificity, and ROC curve analysis.

## INTRODUCTION

The gastric cancer mortality rate remains high in Japan. In 2007, it was the second leading cause of cancer-related death, behind only lung cancer in men and colorectal cancer in women.^1^ However, the rate has been decreasing in recent years, and gastric cancer screening has been cited as one reason for the decrease.^2^^,^^3^ The authors of the “Assessment of Effectiveness of New Screening Techniques for Cancer”^4^ concluded that there exists reasonable evidence that gastric cancer mortality declined after the introduction of mass screening using fluoroscopy.

High-density barium sulfate was recently developed and is used in many medical facilities. Indeed, the 2005 guidelines of the Japanese Society of Gastroenterological Cancer Screening (JSGCS) recommended its use for gastric cancer screening.^5^ There is no standard definition of high-density barium sulfate. Doi et al^6^ suggested a definition of 180 w/v% and 150 ml, and this has been widely accepted. The temperature of the barium, and the pH and volume of gastric juice have little effect on the viscosity of high-density barium sulfate.^7^^,^^8^ Thus it has better acid resistance and fluidity than moderate-density barium sulfate.^9^ As a result, high-density barium sulfate permits superior depiction of the gastric mucosa,^10^^–^^13^ and it is easier for screenees to drink.^9^^,^^13^^–^^15^ Its disadvantages are rapid outflow from the stomach^16^^,^^17^ and a higher incidence of mis-swallowing.^14^^,^^18^ Hamashima et al^19^ conducted a systematic review of existing data and reported that the superiority of high-density barium sulfate could not be confirmed because there had been no improvement in the rate of gastric cancer detection or early gastric cancer detection.^10^^,^^20^^–^^22^ They recommended additional research to clarify the diagnostic validity of the new method.

The aim of the present study was to compare the diagnostic validity of gastric cancer screening using high-density barium sulfate with that of the conventional method using moderate-density barium sulfate, with respect to sensitivity, specificity, and area under receiver-operating-characteristic (ROC) curves.^23^ Linkage of medical records with the Osaka Cancer Registry enabled follow-up of screenees.^24^ Using this system, the diagnostic validity of the gastric cancer screening test was assessed by Murakami et al.^25^ Although evaluations of the diagnostic validity of the gastric cancer screening test have been performed in other prefectures in Japan,^26^^,^^27^ evaluation of the diagnostic validity of screening with high-density barium sulfate has yet to be conducted using area-under-the-curve (AUC) analysis. The present study is the first in Japan to assess the diagnostic validity of gastric cancer screening using high-density barium sulfate.

## METHODS

### Subjects

The protocol for this study was approved by the Ethical Committee of the Osaka Cancer Prevention and Detection Center. The study subjects were 171 833 residents of Osaka, Japan who underwent gastric cancer screening at the Center during the period from 1 January 2000 through 31 December 2002. The subjects were screened by a new method using high-density barium sulfate (*n* = 48 336; conducted at the Center or at mobile units) or by the conventional method using moderate-density barium sulfate (*n* = 123 497; at mobile units).

### Materials and radiographic methods

The moderate-density barium sulfate suspensions used in this study were Barytgen sol 145 w/v% sol, 200 ml (Fushimi Pharmaceutical Co., Ltd., Marukame, Japan) and Baritop sol 150 w/v% sol, 200 ml (Kaigen Co., Ltd., Osaka, Japan). The high-density barium sulfate suspensions were Baribright P 185 w/v% powder, 160 ml (Kaigen Co., Ltd.) and Barytgen HD 200 w/v% powder, 145 ml (Fushimi Pharmaceutical Co., Ltd.). Gastric cancer screening was performed at 10 mobile screening units, and at the Osaka Cancer Prevention and Detection Center, using 2 fluoroscopic devices: the U-MA5N (Hitachi Medical Co., Ltd. Tokyo, Japan) and ZS-40 (Shimadzu Co., Ltd. Kyoto, Japan). Radiographs were obtained using image-intensifier fluorography with 100-mm roll film, namely, Kodak PFH-T FILM (Eastman Kodak Co., Ltd., Rochester, N.Y.) and Fuji MI-FA (Fujifilm Corporation, Kanagawa, Japan).

The new method (high-density barium sulfate) was recommended by the Japan Society of Gastroenterological Cancer Screening (JSGCS).^5^ The images obtained using this method were: (1) double-contrast view in the supine position, (2) double-contrast view (lower) in the prone position, (3) double-contrast view (upper) in the prone position, (4) right anterior oblique position, (5) left anterior oblique position (lower), (6) left anterior oblique position, (7) semi-erect in the left anterior oblique position, and (8) semi-erect in the right anterior oblique position. The images obtained using the conventional method (moderate-density barium sulfate^28^) were: (1) anterior mucosal view in the prone position, (2) barium-filled view in the prone position, (3) double-contrast view in the supine position, (4) left anterior oblique position, (5) right anterior oblique position, (6) semi-erect left anterior oblique position, and (7) barium-filled view in the erect position. The abovementioned 7 or 8 images were used as the basis for the imaging examination; extra images were obtained by the radiological technologists when deemed necessary.

### Screening test and follow-up

The images from gastric cancer screening were examined by 19 radiological technologists and 20 radiologists at the Osaka Cancer Prevention and Detection Center. Images from both radiographic methods were examined by these same radiological technologists and radiologists. A double-check system using 2 radiologists was used for film reading. The radiographic findings were divided into 5 groups: (A) definite cancer, (B) probable cancer, (C) possible cancer, (D) suspected benign lesion, and (E) workup tests for confirmation.^29^

To detect false-negative cases, follow-up was conducted by linking the gastric cancer screening records from the screening center with data from the Osaka Cancer Registry through 31 December 2003. References for individual identification were name, sex, birth date, and address. Cases of cancer detected within 1 year of the screening day were considered as cancer present at the time of screening, and the sensitivity and specificity were calculated.^30^ Sensitivity and specificity were also calculated by sex, by age group (<60, ≥60 years), and for all subjects (both sexes and age groups). The sensitivity and specificity of high-density barium sulfate screening was compared with those of moderate-density barium sulfate screening, and the diagnostic validity of the screening methods was determined.

### Statistical analyses

The Cochran–Mantel–Haenszel test was used to analyze the age and sex distribution of the subjects. The chi-square test was used to analyze the results of the screening, ie, sensitivity and specificity. AUCs were compared using the algorithm developed by DeLong et al.^23^ The diagnostic validity of high-density barium sulfate screening was compared with that of moderate-density barium sulfate screening by using AUC values. A value of *P* < 0.05 was considered statistically significant. These statistical analyses were performed using SPSS 15.0 J for Windows (SPSS Japan Inc., Tokyo, Japan). AUCs for the 2 barium sulfate screening methods were analyzed using Stata 9.2 for Windows (StataCorp LP, College Station, TX).

## RESULTS

Table 1 shows the sex and age distributions of the screenees in the high- and moderate-density barium sulfate tests. The male/female ratio was greater than 1 in the high-density test, and less than 1 in the moderate-density test. The mode for age in the high-density test was 50–59 years among both men and women; in the moderate-density test, the mode for age was 50–59 years among women and 60–69 years among men. There were significant differences in sex and age distributions between screenees in the 2 groups (*P* < 0.05).

**Table 1.** Sex and age distribution of screenees

Age (yrs)	High-density barium sulfate screening				Moderate-density barium sulfate screening			
	
	Male (%)		Female (%)		Male (%)		Female (%)	
<29	68	(0.2)	46	(0.2)	79	(0.2)	40	(0.1)
30–39	3246	(11.1)	875	(4.6)	2129	(4.4)	3194	(4.2)
40–49	9760	(33.4)	4681	(24.5)	8845	(18.3)	12 573	(16.7)
50–59	9977	(34.1)	6771	(35.4)	12 449	(25.8)	26 966	(35.8)
60–69	4618	(15.8)	5131	(26.9)	17 538	(36.4)	25 903	(34.4)
70–79	1371	(4.7)	1485	(7.8)	6618	(13.7)	6146	(8.2)
>80	183	(0.6)	124	(0.7)	588	(1.2)	429	(0.6)
Total	29 223	(100.0)	19 113	(100.0)	48 246	(100.0)	75 251	(100.0)

Cochran–Mantel–Haenszel test.

There were significant differences in the age and sex distributions of the 2 test groups (*P* < 0.05).

A comparison of the screening results for the 2 groups is shown in Table 2. The proportion of screenees for whom a workup examination was recommended (screening positives) was lower for high-density than for moderate-density barium sulfate screening (8.7% vs 9.2%, respectively; *P* = 0.001). The proportion of screenees who underwent workup examinations was 86.8% for the moderate-density barium sulfate test and 85.6% for the high-density test (*P* = 0.044). The gastric cancer detection rate was 0.17% for moderate-density barium sulfate and 0.13% for high-density barium sulfate, and the proportion of early gastric cancer detected was 67.6% in the former and 62.9% in the latter.

**Table 2.** Results of screening in the 2 screening groups

Result	High-density (%)		Moderate-density (%)		*P* value
No. of screenees	48 336		123 497		
Recommended for ​ workup tests	4201	(8.7)	11 341	(9.2)	0.001
Underwent workup tests	3596	(85.6)	9849	(86.8)	0.044
Gastric cancer detected	62	(0.13)	207	(0.17)	0.064
Early gastric cancer ​ detected	39	(62.9)	140	(67.6)	0.489

Chi-square test.

High-density: High-density barium sulfate.

Moderate-density: Moderate-density barium sulfate.

Table 3 shows the linkage of the results of follow-up in both groups with the records of the Osaka Cancer Registry; Table 4 shows the results of follow-up with respect to sex and age (≥60, <60 years). All false-negative cases (6 cases from the high-density test and 19 cases from the moderate-density test) were detected during follow-up using record linkage to the cancer registry. Some true-positive cases (5 cases from the high-density test and 20 cases from the moderate-density test) were not identified by routine collection of data regarding examination work-ups, but were discovered for the first time as a result of record linkage to the cancer registry.

**Table 3.** Results of follow-up for all subjects

Screening		Cancer		Total

		Present	Absent	
High-density barium sulfate	Positive	67	4134	4201
	Negative	6	44 129	44 135
	Total	73	48 263	48 336
Moderate-density barium sulfate	Positive	227	11 114	11 341
	Negative	19	112 137	112 156
	Total	246	123 251	123 497

Period: From January 1, 2000 through December 31, 2002.

**Table 4.** Screening result and cancer status on follow-up, by sex and age

Screening		Sex						Age					
	
		Male			Female			≥60 yrs			<60 yrs		
			
		Present	Absent	Total	Present	Absent	Total	Present	Absent	Total	Present	Absent	Total
High-density barium sulfate	Positive	53	2640	2693	14	1494	1508	42	1439	1481	25	2695	2720
	Negative	5	26 525	26 530	1	17 604	17 605	1	11 430	11 431	5	32 699	32 704
	Total	58	29 165	29 223	15	19 098	19 113	43	12 869	12 912	30	35 394	35 424
Moderate-density barium sulfate	Positive	146	5580	5726	81	5534	5615	181	6169	6350	46	4945	4991
	Negative	9	42 511	42 520	10	69 626	69 636	14	50 858	50 872	5	61 279	61 284
	Total	155	48 091	48 246	91	75 160	75 251	195	57 027	57 222	51	66 224	66 275

Period: From January 1, 2000 through December 31, 2002.

Table 5 shows the test performance values for screening with the 2 densities of barium sulfate among all subjects. The predictive value of positive tests was 2.00% for moderate-density barium sulfate and 1.59% for high-density barium sulfate. Although there was no significant difference in sensitivity (92.28% for moderate-density barium sulfate vs 91.78% for high-density barium sulfate), there was a significant difference in specificity (90.98% vs 91.43%; *P* = 0.003). Table 6 shows the sensitivity and specificity of the 2 tests with respect to sex and age group. There was no significant difference in sensitivity in any comparison. Although the specificity of the 2 tests significantly differed in both men and women (*P* < 0.001 and *P* = 0.031, respectively), it did not significantly differ by age group.

**Table 5.** Performance values of screening in the 2 screening groups

	High-density	Moderate-density	*P* value
Sensitivity (%)	91.78	92.28	0.890
Specificity (%)	91.43	90.98	0.003

Chi-square test.

High-density: High-density barium sulfate.

Moderate-density: Moderate-density barium sulfate.

**Table 6.** Performance values of screening in the 2 test groups, by sex and age

	Sex						Age					
		
	Male			Female			≥60 yrs			<60 yrs		
				
	High	Moderate	*P* value	High	Moderate	*P* value	High	Moderate	*P* value	High	Moderate	*P* value
Sensitivity (%)	91.38	94.19	0.461	93.33	89.01	0.611	97.67	92.82	0.236	83.33	90.20	0.365
Specificity (%)	90.95	88.40	<0.001	92.18	92.64	0.031	88.82	89.18	0.231	92.39	92.53	0.397

Chi-square test.

High: High-density barium sulfate.

Moderate: Moderate-density barium sulfate.

**Figure 1.**
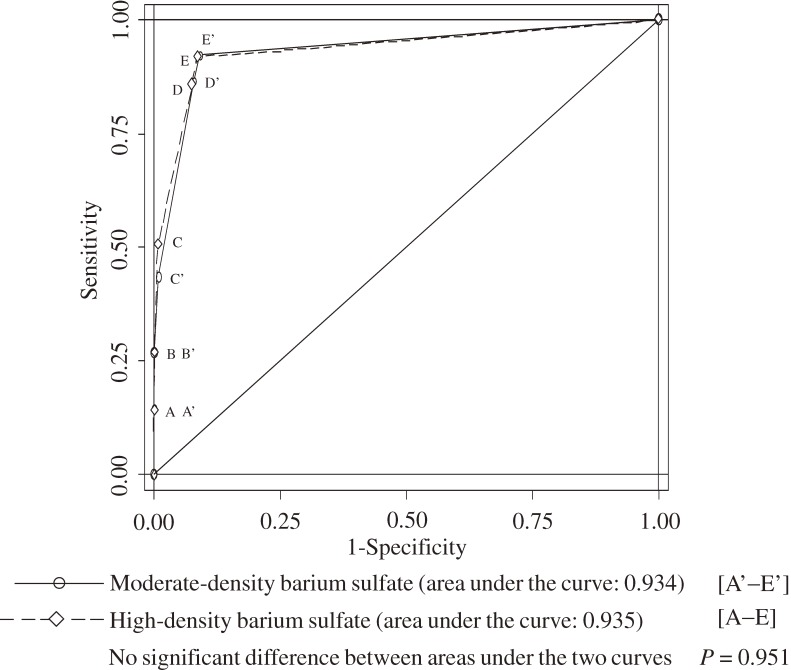
Area under ROC curve (AUC) analysis of tests using high- and moderate-density barium sulfate. Degree of cancer suspicion: A, definite cancer; B, probable cancer; C, possible cancer; D, suspected benign lesion; E, workup test for confirmation.

Table 7 shows the sensitivity and specificity of the 2 tests when the cut-off point was adjusted according to the degree of cancer suspicion (A, B, C, D, E). When using high-density barium sulfate, the sensitivities were A: 13.70%, B: 27.40%, C: 50.68%, D: 84.93%, and E: 91.78%; the specificities were A: 99.99%, B: 99.94%, C: 99.18%, D: 92.58%, and E: 91.43%. When using moderate-density barium sulfate, the sensitivities were A: 14.23%, B: 26.83%, C: 43.50%, D: 86.18%, and E: 92.28%; the specificities were A: 99.99%, B: 99.93%, C: 99.17%, D: 92.19%, and E: 90.98%. The AUC value for the high-density barium sulfate test (0.935) was slightly higher than that for the moderate-density barium sulfate test (0.934); however, the difference was not significant (*P* = 0.951; Figure 1).

**Table 7.** Sensitivity and specificity of the 2 barium sulfate screening tests, by degree of cancer suspicion

Cut-off point	High-density barium sulfate		Moderate-density barium sulfate	
	
	Sensitivity (%)	Specificity (%)	Sensitivity (%)	Specificity (%)
A	13.70	99.99	14.23	99.99
B	27.40	99.94	26.83	99.93
C	50.68	99.18	43.50	99.17
D	84.93	92.58	86.18	92.19
E	91.78	91.43	92.28	90.98

Degree of cancer suspicion: A, definite cancer; B, probable cancer; C, possible cancer; D, suspected benign lesion; E, workup tests for confirmation.

## DISCUSSION

In the present study, the proportion of screenees for whom a workup examination was recommended (screening positives) was smaller for the high-density barium sulfate test than for the moderate-density barium sulfate test. Specificity was higher for the high-density barium sulfate test, but the difference was not significant; sensitivity did not significantly differ between the 2 tests. The 2 tests also did not differ in diagnostic validity, which was measured by using AUC values.

The diagnostic validity of the screening tests was examined by follow-up with record linkage to a cancer registry. Cases of cancer detected within 1 year of the screening day were classified as cancer present at the time of screening, and the sensitivity and specificity were calculated.^31^ Sensitivity and specificity are optimal measures for analyzing diagnostic validity, and are not dependent upon the prevalence of cancer. In addition, the ability to detect gastric cancer on radiographs has been reported by Yatake et al^31^ using AUC analysis. The present study is the first to compare the sensitivity, specificity, and AUC values of tests that used 2 different densities of barium sulfate.

Previous studies of high-density barium sulfate revealed that it has a wider double-contrast range than that of conventional moderate-density preparations, which results in superior depiction of gastric cancer due to better barium adherence.^13^ Furthermore, inadequate adhesion of barium to the gastric cardia and fornix^32^ can be overcome using high-density barium sulfate.^10^^,^^13^ Many studies have reported that high-density barium sulfate improves imaging of the gastric mucosa.^10^^,^^33^^–^^38^ In addition, because of its low viscosity and the small quantity required, it is easy for screenees to drink.^9^^,^^13^^–^^15^^,^^34^^,^^39^^,^^40^ Moreover, as compared with moderate-density barium sulfate, its viscosity is more stable despite changes in barium temperature and alterations in the pH and volume of gastric juice.^7^^,^^8^ In addition, the fluidity of high-density barium sulfate is excellent on fluoroscopic monitors.^9^

Agglutination was found to be greater in high-density than in moderate-density barium sulfate in 3 reports,^10^^,^^37^^,^^38^ equal in 1 report,^11^ and lower in 2 reports.^9^^,^^35^ Outflow of barium sulfate from the stomach was faster for high-density than moderate-density barium sulfate in 2 reports^16^^,^^17^ and equal in 2 reports,^11^^,^^36^ 1 report noted no outflow.^13^ No significant difference in gastric cancer detection rate^19^ was observed in screening tests using high- and moderate-density barium sulfate preparations.^10^^,^^20^^–^^22^^,^^41^^,^^42^ Regarding early gastric cancer detection rate, 4 articles reported no significant difference^10^^,^^20^^–^^22^ between the 2 preparations, and 3 articles reported a significantly higher rate for high-density barium sulfate.^41^^–^^43^ Yamamoto et al found no significant difference in radiation dose between screenings conducted using high- and moderate-density barium sulfate.^44^^,^^45^ It has been reported that men aged 80 years or older have the highest risk of mis-swallowing.^14^^,^^18^ No significant difference in discharge time or conditions has been observed between the 2 barium sulfate densities.^13^^,^^15^^,^^46^ Hamashima et al reported, “We could not find any evidence on the excellence of gastric cancer screening using high-density barium sulfate from the systematic review. Further appropriate research is obviously required to clarify the diagnostic validity of the new method.”^19^ The present study was the first in Japan to compare the sensitivity, specificity, and AUC for a gastric cancer screening test that used high-density barium sulfate.

The present study has some limitations. First, the study was observational and the subjects were not randomly assigned to undergo screening with high-density or moderate-density barium sulfate. There was a significant difference in the sex and age distributions of screenees in the 2 test groups. When screenees were stratified by sex and age group, sensitivity was higher for moderate-density than for high-density barium sulfate in men, whereas the reverse was true for women. Sensitivity was higher for the moderate-density barium sulfate test among screenees younger than 60 years, and higher for the high-density barium sulfate test among screenees 60 years or older; however, these differences in sensitivity were not significant. These findings do not contradict the conclusions of the present study. A significant difference was observed in specificity, but the individual values for specificity did not greatly differ. Sex and age group trends were much the same as those for all subjects. The significant difference observed in specificity between the 2 tests may have arisen because of the very large number of subjects. Briefly, the large sample size narrows the confidence interval to a significant difference. In addition, specificity alone has little clinical importance; evaluation of diagnostic validity should be based on both sensitivity and specificity. Second, the results of the 1-year follow-up with record linkage to the Osaka Cancer Registry were defined as the gold standard in the present study. However, the completeness of the Osaka Cancer Registry should be treated with caution, and a second standard should be developed. Nonetheless, we believe that comparability between the 2 barium sulfate tests was retained because the same follow-up methods were used and because of the criteria for false negatives in the 2 groups. Third, it is very important to guarantee the comparability of the 2 groups when comparing testing methods. In this study, differences in the diagnostic ability of the 19 radiological technologists and 20 radiologists were not assessed, and comparability thus cannot be guaranteed. However, the same radiological technologists performed radiography using both the new and conventional methods, the same radiologists read the radiographs, and the percentages of those who were involved in the examinations using both methods were nearly the same; therefore, the effect of differences in diagnostic ability on the results of this study would likely be small.

We believe that the results reflect the new method’s superior depiction ability and fast passage of barium; however, because of the small volume of barium swallowed and fast outflow from the stomach, there may be less barium in the stomach with the new high-density barium sulfate method. Therefore, the radiographic technologists are required to rapidly turn and roll the screenees to obtain the films before the barium is lost. It will therefore require more study before we can establish whether the diagnostic validity of the new method is better than that of the conventional method.

## CONCLUSION

There was no significant difference in specificity, sensitivity, or AUC between tests using 2 different densities of barium sulfate. The screening tests with high-density and moderate-density barium sulfate had similar validity in terms of sensitivity, specificity, and ROC curves. These results indicate that the moderate- and high-density barium sulfate tests can both be recommended for use in X-ray screening for gastric cancer.
